# Behavioral manifestations in rodent models of autism spectrum disorder: protocol for a systematic review and network meta-analysis

**DOI:** 10.1186/s13643-022-02028-w

**Published:** 2022-07-26

**Authors:** Alana Castro Panzenhagen, Amanda Cavalcanti, Dirson João Stein, Ligia Lins de Castro, Mailton Vasconcelos, Mariana Boechat Abreu, Roberto Farina Almeida, Leandro José Bertoglio, Ana Paula Herrmann

**Affiliations:** 1 Brazilian Reproducibility Initiative in preclinical Systematic Review and Meta-Analysis (BRISA) Collaboration, Rio de Janeiro, RJ Brazil; 2 Programa de Pós-Graduação em Ciências Biológicas: Bioquímica, Universidade Federal do Rio Grande do Sul, Porto Alegre, RS Brazil; 3 Instituto Nacional de Traumatologia e Ortopedia, Rio de Janeiro, RJ Brazil; 4 Programa de Pós-Graduação em Medicina: Ciências Médicas, Universidade Federal do Rio Grande do Sul, Porto Alegre, RS Brazil; 5 Instituto de Biofísica Carlos Chagas Filho, Universidade Federal do Rio de Janeiro, Rio de Janeiro, RJ Brazil; 6 Faculdade de Medicina, Universidade Federal do Rio Grande do Sul, Porto Alegre, RS Brazil; 7 Departamento de Ciências Biológicas, Programa de Pós-Graduação em Ciências Biológicas, Universidade Federal de Ouro Preto, Ouro Preto, MG Brazil; 8 Departamento de Farmacologia, Universidade Federal de Santa Catarina, Florianópolis, SC Brazil; 9 Departamento de Farmacologia, Instituto de Ciências Básicas da Saúde, Universidade Federal do Rio Grande do Sul, RS Porto Alegre, Brazil

**Keywords:** Animal model, Autism, Autism spectrum disorder, Rodent model, Systematic review, Network meta-analysis, Protocol

## Abstract

**Background:**

Autism spectrum disorder (ASD) is a neurodevelopmental condition associated with severe social communication, interaction, and sensory processing impairments. Efforts to understand its etiology and pathophysiology are crucial for improving treatment and prevention measures. Preclinical models of ASD are essential for investigating the biological mechanisms and should present translatability potential. We aim to evaluate the consistency of the most commonly used rodent models of ASD in displaying autistic-like behavior through a systematic review and meta-analysis.

**Methods:**

This review will focus on the most frequently used autism models, surveying studies of six genetic (*Ube3a*, *Pten*, *Nlgn3*, *Shank3*, *Mecp2*, and *Fmr1*), three chemically induced (valproic acid (VPA), lipopolysaccharide (LPS), and polyinosinic:polycytidylic acid (poly(I:C))), and one inbred model (BTBR T+ Itpr3tf/J mouse strain). Two independent reviewers will screen the records. Data extraction of behavioral outcomes and risk of bias evaluation will be performed. We will conduct a meta-analysis whenever at least five studies investigate the same model and behavioral outcome. We will also explore the heterogeneity and publication bias. Network meta-analyses are planned to compare different models.

**Discussion:**

By shortening the gap between animal behavior and human endophenotypes or specific clinical symptoms, we expect to help researchers on which rodent models are adequate for research of specific behavioral manifestations of autism, which potentially require a combination of them depending on the research interest.

**Systematic review registration:**

PROSPERO CRD42021226299.

**Supplementary Information:**

The online version contains supplementary material available at 10.1186/s13643-022-02028-w.

## Background

Autism spectrum disorder (ASD), or simply autism, is a neurodevelopmental condition characterized by severe impairments in social communication, interaction, and sensory processing, often accompanied by repetitive behaviors and restricted interests. At the most severe level, patients with ASD may also present varying levels of intellectual disability. Moreover, attention deficit/hyperactivity, anxiety, major depressive disorders, and epilepsy are relatively frequent comorbidities in people with autism, making their therapeutic management further challenging [1, 2].

The worldwide prevalence of autism is below 1% [3], yet its diagnosis and identification have dramatically increased in the last decades. Epidemiological studies indicate significant variability in prevalence globally, although it is remarkably higher in high-income countries. ASD is highly heritable, occurs in all ethnic and socioeconomic groups, and is over four times more common among males than among females [4].

Despite extensive clinical and preclinical studies, the etiology and pathogenesis of ASD remain unclear. It has gradually led scientists to use in vitro and in vivo animal models to uncover the causes of ASD and improve treatment. This endeavor provided advances in understanding ASD pathophysiology, shedding light on new targets for therapy. Overall, animal models rely on a single gene dysfunction, epigenetic manipulations, or environmental interventions that ultimately influence the expression of risk genes. Although the behavioral assays mimicking specific symptoms are excellent translational research tools to investigate and identify the biological mechanisms underlying the core features of ASD [5], there has been no systematic investigation on whether they are interchangeable or complement each other. Considering the heterogeneity and complexity of ASD, it is hypothesized that a combination of various animal models is necessary to recapitulate its main behavioral manifestations. As a result, compiling information by reviewing and comparing existing data may more effectively guide future researchers’ efforts.

Three criteria have been considered for assessing the validity of a given animal model, namely, face validity (i.e., does the model exhibit the salient features of the condition in humans?), construct validity (i.e., is the condition arising from the same biological background?), and predictive validity (i.e., will the model respond to well-established treatments?) [6]. Das et al. [7] present a manually curated annotation tool that used to be updated quarterly and gathered information on ASD research for circa 10 years. AutDB (https://gene.sfari.org/database/animal-models/genetic-animal-models/) is a database platform. In 2019, as reported by Das et al., there were 787 articles identified and 18 behavioral phenotypes most frequently evaluated in genetic, induced, and inbred ASD rodent models. These behaviors were classified as core and auxiliary. Core behaviors are represented by social interaction, ultrasonic vocalization, and repetitive behavior. This classification was proposed by Basu et al. [8] when creating AutDB based on their similarity to the human phenotype (impairment related to social interaction, communication, and repetitive behavior). Das et al. [7] (from the same research group) provide an update regarding the annotated data so far. In order to summarize the evidence from the original studies annotated in AutDB, the researchers separated the results into qualitative terms to indicate the direction of change compared to control animals. Regarding the core categories, “no change” represented at least 36% of the annotations in the autism models used in the analyzed articles. In the auxiliary categories, the lack of changes was even more expressive, reaching 70%.

AutDB is a manually curated tool; however, its methodology for search strategy and selection of studies is far from a systematic review. Moreover, the platform started with a focus on genes for ASD. The search consisted of the terms “gene” AND (“autism” OR “autistic”) restricted to the titles and abstracts of the publications for retrieval, only on PubMed. Therefore, we understand that the literature on ASD-like animal models is expressively larger than the one reported in AutDB. Furthermore, there is no quality control or assessment of the risk of bias in the studies annotated, nor is there any data extraction process for conducting meta-analyses. In view of this gap, and considering that Basu et al. [8] and Das et al. [7] are the only reports on attempts to gather the literature on ASD-like animal models, we intend to conduct a thorough systematic review to evaluate the validity and compare different rodent models of ASD available to date. Nonetheless, we based our inclusion criteria on Das et al. [7] report to choose the likely most frequently used models and evaluated behaviors.

This review aims to test model face validity and assess whether the most commonly used rodent ASD models reproduce behavioral phenotypes related to the core symptoms of the condition in humans. Moreover, we mean to understand what secondary phenotypes are also altered in such models. Finally, we will hopefully show which rodent models are more suitable for research in this area, enabling the assertion of models or compilation of models that present specific behavioral manifestations of ASD.

## Methods

The Preferred Reporting Items for Systematic Review and Meta-Analysis Protocols (PRISMA-P) statement [9] was followed to elaborate this protocol, which was registered in PROSPERO under registration number CRD42021226299. We intend to answer the following question through this study: what are the differences and similarities between the behavioral manifestations commonly assessed in the most widely used induced, inbred, or genetic rodent ASD models?

### Model selection

This review will focus on the most frequently used ASD rodent models according to the estimate by Das et al. [7], which was based on data retrieved from AutDB. This comprehensive and integrated database collates in-depth annotation of genetic and non-genetic ASD models [8]. A frequency cutoff of at least ten references of either mouse or rat studies was set, leading to the selection of six genetic model groups based on the manipulated genes (*Ube3a*, *Pten*, *Nlgn3*, *Shank3*, *Mecp2*, and *Fmr1*), three chemically induced models (valproic acid (VPA), lipopolysaccharide (LPS), and polyinosinic:polycytidylic acid (poly(I:C))), and one inbred model (BTBR T+ Itpr3tf/J mouse strain). The number of references refers directly to what is reported in Das et al. [7], not necessarily corresponding to the number of studies or experiments. This corresponds to the “number of reports” in AutDB (https://gene.sfari.org/database/animal-models/genetic-animal-models/). This evidence has been used to choose the models to be included in the present systematic review. However, none of the data will be retrieved from the annotation tool. A full systematic review will be performed since the methodology used in AutDB does not compare to it in terms of criteria, accuracy, or inclusion of the whole literature on the matter.

### Eligibility criteria

We will include preclinical studies evaluating behavioral outcomes in selected ASD models in mice and rats (*P-participants/individuals*). The *E-exposure* will include alterations of the whole organism at the DNA level for the genetic model groups based on the manipulated genes, without restriction for temporally controlled conditional models. We will include genetic alterations, such as knock-down, knock-in, or heterozygous models. In this sense, we will group the models as to the risk gene function (increased or decreased gene expression). Although we are confident there will be enough studies by model (we expect to find more studies through a systematic review than AutDB did), we also would like to see the results by groupings, which would make sense due to their biological nature. However, if heterogeneity is too high because of the variability in model development, we will refrain from making any conclusions from these analyses. Only interventions (*E-exposure*) administered prenatally or postnatally until weaning day (PND21) will be included for the induced models. Only studies with a comparison group (*C-comparison*) will be included; these will include control (without any intervention), sham (same staged intervention with a vehicle application or no actual induction), and wild-type (background genetic architecture). Any behavioral (*O-outcome*) related to the following broad categories will be considered: social or repetitive behavior, communication, emotion, learning and memory, and sensory and motor function (seizure-related behavior will not be included). There will be no date or language restrictions.

Any publication that does not include original data will be excluded (e.g., review, letter, editorial, comment). We will also exclude studies (1) conducted in species other than rats or mice or purely ex vivo, in vitro, or in silico; (2) without a comparison group (model control); (3) using cell- or tissue-specific genetic models; (4) in which VPA, LPS, or poly(I:C) was not administered prenatally or postnatally until the weaning day (or PND21); (5) in which the groups of interest were subjected to an additional experimental intervention (e.g., stress, rescue treatment); and (6) lacking the report of a behavioral outcome in the categories of interest.

### Search strategy

Studies will be identified through a literature search using three electronic databases: MEDLINE, Web of Science Core Collection, and Scopus. The basic combination of search concepts consists of (((Genetics or strain ASD model terms) OR ((Neonatal developmental terms) AND (Induced ASD model terms))) AND (Animal model identification terms). The detailed combination of search terms constructed for each database is shown in Additional file 1. No search filters are going to be used.

### Report identification and selection

The identification and selection of reports (both for abstract and full-text screening) will be performed by nine reviewers using a web-based/smartphone application systematic research tool: Rayyan (Rayyan Systems Inc.). After gathering the search results from the three selected databases, duplicates/triplicates will be removed manually with Rayyan’s automatic suggestions. Next, the list of unique hits will be randomly split into sets. Each set will be evaluated by a pair of independent reviewers, with a third reviewer as a tiebreaker for conflict cases, ensuring that at least two reviewers evaluate each identified report. The teams of reviewers will be formed at random, and each one will be combined with at least four other reviewers in four sets of unique studies to guarantee diversity in the decision-making style of the report selection. As reviewers will also serve as tiebreakers in other groups, meetings will be held regularly throughout the selection process to discuss identified error biases or address specific cases; this approach will unify decision rules across all reviewers.

The report selection and inclusion will be performed with a two-step screening process. Initially, the decision will be based on the title and abstract, and, if necessary, a full-text screening will be performed to include a report. The full-text review step will also be performed if an exclusion criterion is not observed in the first step.

### Data collection

Five reviewers will extract all data, and a training procedure will be implemented to assure standardization of the process. The quality control procedures will be implemented in the early extraction stages. During the pilot phase of data collection, we expect to determine common variables for the more frequent phenotypic analysis, reducing data amount and facilitating the training for homogeneous and reliable data extraction. When the information required is not available, the corresponding author or the first and last authors of the original studies will be e-mailed. If no answer is received in 2 months, the records will be excluded from the analysis. Data and tables with relevant information will be accessed manually. Information of graphs and figures will be extracted using the WebPlotDigitizer version 4.3 software.

The following data will be extracted: study design (controlled trial or cross-over, number of experimental groups, and sample sizes) and animal model (species, strain, sex, type of disease induction, age of the animal upon induction, age at measurements, number of control groups, type of housing after weaning, and outcome assessment). In the case of induced models, intensity, dose, and administration route will be assessed. All reported measures will be extracted for the tests described in the “Outcomes” section.

The summary statistics to be extracted are mean, standard deviation (SD) by group, sample size when data are continuous, or percentage and sample size when data are dichotomous. Other effect measures will be extracted when the mean and SD or percentage are not available. We will recheck a random sample of 10% of studies and, based on the results, cross-check all the remaining data only for the variables where the highest proportion of mistakes are observed (“tricky variables”).

### Outcomes

A complete profile of behavioral outcomes in rodent ASD models adapted from AutDB will be used in this study. We will include tests of social memory, social interaction, social approach, self-grooming, repetitive digging, ultrasonic vocalization, exploratory activity, anxiety, spatial reference memory, spatial learning, object recognition memory, cued or contextual fear conditioning, motor coordination and balance, general locomotor activity, startle response, sensorimotor gating, and pain or nociception. A complete description of each behavioral category is listed in Additional file 2, where the phenoterms and tests, as described by Das et al. [7], are classified into seven categories (social behavior, repetitive behavior, communications, emotions, learning and memory, sensory, motor). In ASD models with face validity, we would expect the following differences (as compared to control animals) in the core behaviors: reduced social behavior (e.g., three-chamber sociability test: time in the social chamber), reduced communication (e.g., ultrasonic vocalizations: number of calls), and increased repetitive behavior (e.g., home cage behavior: repetitive rearing and climbing). The evaluation of outcomes from the auxiliary categories of phenoterms (emotion, learning and memory, sensory, motor) will be exploratory for the different studied models.

### Risk of bias

Assessment of risk of bias in included studies will be evaluated by the SYstematic Review Centre for Laboratory animal Experimentation (SYRCLE’s) risk of bias tool (RoB) [10], with suitable modifications adjusted by aspects that play relevant roles in ASD-associated rodent models. Each report criterion of the SYRCLE RoB tool to detect the risk of bias will be judged by experienced investigators according to the following items: (1) reporting of random allocation, (2) reporting of baseline characteristics, (3) reporting regarding if the animals were randomly housed during the experiment, (4) reporting the blinding methods used by caregivers and investigators, (5) reporting animal random outcome assessment, (6) reporting of blinded assessment of outcome, (7) reporting of animal exclusions, (8) selective outcome reporting, (9) reporting the correct unit-of-analysis, and (10) reporting of sample size calculation. Classification of low, high, or unclear risk of bias will be assigned for each item evaluating every included report, except the first item (*reporting of random allocation*) that will be characterized as low risk of bias (authors describe the method used to randomize), unclear (authors only say “random” without any specification), uncertain (authors did not describe the method used to randomize the sample), and high risk of bias (it is not random). After the risk of bias assessment, we will recheck a random sample of 10% of reports. Tricky items will be considered and discussed when interpreting the results.

### Data synthesis

A meta-analysis will be conducted whenever at least five studies have the same design, reporting data for the same animal model, comparison group, and behavioral test type. The effect measures used to perform the meta-analysis will be standardized mean difference and odds or risk ratios when the first is impossible. The analysis will follow a random-effects model to account for heterogeneity. We will use “report” as a random factor. Notwithstanding, *I*^2^ and Cochran *Q* statistics will be employed to quantify the statistical heterogeneity among studies. We will investigate any possible source of heterogeneity after conducting a subgroup analysis and consider adding them to the random-effects model.

Whenever one control is used multiple times, the final sample (of that control group) will be adjusted by dividing the total sample size by the number of times that group is included in the analysis.

A network meta-analysis will also be performed, comparing the models for each outcome, provided that each model has at least five different studies investigating the same behavior. Subgroup meta-analyses (meta-regression or stratified regression) will also be conducted according to the following potential heterogeneity introducing variables: species, strain, sex, intensity and duration of model behavior induction, age and weight of animals, lab/study group, and analyses by specific behavioral tests. All these will be performed when the subgroup is composed of at least ten different original studies.

When there are multiple and comparable outcomes reported for the same behavioral test (e.g., for elevated plus-maze: time spent in open arms, distance traveled in open arms, number of entries in open arms), we will choose the most frequently reported metric across studies; if a report does not report the chosen metric, then we will use the second most frequently reported metric, and so forth. The sign of the effect size will be reversed (multiplying it by minus one) when needed so that the direction of the effect can be interpreted consistently if the metrics have opposite meanings for the behavioral trait (e.g., exploration in the open versus closed arms in the elevated plus-maze; correct choices versus the number of errors in learning tests). Behavioral variables that are not the most common across reports for the same behavioral test will not be used in the meta-analysis. Moreover, we will refrain from mixing different behavioral tests that are not based on the same apparatus (e.g., grooming in the open field test and grooming in the elevated plus-maze test).

Sensitivity analyses will be performed as follows: (a) following the Jackknife method for all main meta-analysis groups, (b) according to the risk of bias quality score of original studies (poorly classified studies with two or more items rated as high risk of bias will be excluded—this represents around a quarter of the studies based on our pilot data), and (c) in case of doubts regarding the assumptions and interpretation of previous analyses. Publication bias will be investigated through funnel plotting and Egger’s regression test [11]. These will only be conducted whenever at least five (for Funnel plots) or ten (for Egger’s regression) studies evaluating the same outcome are available.

All analyses will be conducted using the R Project for Statistical Computing (https://www.r-project.org/) [12] packages *metafor* (https://cran.r-project.org/package=metafor) [13] and *ggplot2* [14].

Whenever a meta-analysis is not possible to be conducted, the descriptive summary and effect sizes from the original studies will be compared qualitatively, also following the five (5) studies’ rule. We will summarize the effect estimates, discussing the range and distribution of observed effects when a comparable estimate of effect is provided (or can be arrived upon through conversion). A narrative summary will be done carefully considering the study quality (including the risk of bias) and sample sizes. Figures (including scatterplots, barplots, or radar plots) will also be constructed to visualize the differences in effect, *P*-value, and direction of findings over the years.

### Piloting

Every step of the methods for this protocol either has been piloted or is planned to be piloted in the next steps of the systematic review. The search described in Additional file 1 and performed on November 5, 2020, identified 18,336 reports in Scopus, 14,202 in Web of Science, and 17,648 in PubMed. The duplicates were removed manually with the help from the Rayyan AI for identification. After this step, the remaining reports (24,983) were randomized, and a sample of 378 reports was used for report selection piloting. Agreement between all nine reviewers reached 95%. Moreover, 6% of the reports were included, leading us to estimate around 1500 to be included in the systematic review.

We have decided to include a variety of behavioral tests, as described in Additional file 2. However, only studies within the same animal model and that have data on the same variables for the same behavioral tests will be compared in the traditional meta-analysis. This means that we have to identify all common tests before going further. With this in mind, the first step in our data extraction procedure will be a screening of all included studies to identify tests and variables used in the original reports. This will guide us as to which studies can be compared. From the screening, we will have data on which animals were used for which behavioral assessment and if the same variables were reported within and between different reports. This will allow us to identify and control the analyses (if applicable) for duplicated samples, repeated measures, and which outcomes are more widely reported for each comparable behavioral assay.

The entire protocol has been based on small pilot studies. A large part of the group has been trained to conduct systematic reviews with meta-analyses of preclinical studies; pilots are the form of training we have adopted. We had pilot phases that often consisted of familiarization, followed by training, and finally, a comparison of reliability or other metrics among researchers. Researchers met regularly to discuss the issues and misunderstandings. That happened for the use of Rayyan and initial selection of the studies and will happen for the data extraction. For the screening pilot, four rounds of judgment were performed with 50–60 hits of random abstracts per round; agreement between all nine reviewers reached 95%.

For the risk of bias analysis pilot, the SYRCLE’s tool for risk of bias assessment was adapted for the two groups of studies based on the induced or genetically altered models of ASD. After three rounds of judgment with 20 full-text studies per round, we obtained an agreement of 85%, on average, for studies of induced models. Training for risk of bias in models based on genetic alterations, data extraction, and conducting meta-analyses has not yet been completed.

## Discussion

We established the current protocol to synthesize and compare the behavioral outcomes of studies using common genetic- (*Pten*, *Fmr1*, *Ube3a*, *Nlgn3*, *Shank3*, or *Mecp2*) and chemically induced (VPA, LPS, or poly(I:C)) rodent models of ASD, besides the BTBR mice, an inbred strain naturally expressing manifestations similar to the core human phenotypes [15]. It is anticipated that researchers interested in this field, especially those aiming at combining complementary models to advance the neurobiology and therapeutic interventions for autism spectrum disorders in humans, will benefit from having comprehensive information on this subject to plan new study designs. Another expected contribution of this review is improving research reproducibility and translatability, minimizing research costs and waste. Finally, by intending to bridge the gap between animal behavior and human endophenotypes or specific clinical symptoms, we expect to foster clinical ASD research indirectly.

## Supplementary Information

**Additional file 1.** Search strategy. The complete search strategy syntax for PubMed, Web of Science, and Scopus databases.

**Additional file 2.** Behavioral outcomes. List of behavioral categories and examples of experimental paradigms.

## Data Availability

All raw data, processed and meta-data, analysis codes used, lists of included and excluded full-text studies, and the datasets generated and analyzed during the current study will be made available in the Open Science Framework repository (10.17605/OSF.IO/DXMW7) [15].

## Abbreviations

ASD: Autism spectrum disorder
PRISMA-P: Preferred Reporting Items for Systematic Review and Meta-Analysis Protocols
Ube3a: Ubiquitin-protein ligase E3A
Pten: Phosphatase and tensin homolog
Nlgn3: Neuroligin-3
Shank3: SH3 and multiple ankyrin repeat domains 3
Mecp2: Methyl CpG binding protein 2
Fmr1: Fragile X mental retardation 1
LPS: Lipopolysaccharide
VPA: Valproic acid
Poly(I:C): Polyinosinic:polycytidylic acid
BTBR: BTBR T^+^Itpr3t^f/J^ mouse strain
PND: Postnatal day
SYRCLE: SYstematic Review Centre for Laboratory animal Experimentation
RoB: Risk of bias
SD: Standard deviation
