# Supplementary material for: Behavioral manifestations in rodent models of autism spectrum disorder: protocol for a systematic review and network meta-analysis
Source: Syst Rev. 2022 Jul 26;11:150. doi: 10.1186/s13643-022-02028-w (PMC9327140; doi:10.1186/s13643-022-02028-w)
Supplement: Supplementary file 1 — Additional file 1. Search strategy. The complete search strategy syntax for PubMed, Web of Science, and Scopus databases. [file 13643_2022_2028_MOESM1_ESM.pdf]

Additional file 1: Search strategy

Search terms were combined with ‘OR’ and concepts were combined with ‘AND’. Queries for the MEDLINE/Pubmed, Web of Science/Clarivate Analytics and Scopus/Elsevier databases are presented below.

Database/Platform	MEDLINE/PubMed
Limits	in: “all fields”<br>date range: all years
Search date and number of hits	05/nov/2020 09:00 am - 17,630
Search query	(((Ube3a OR “ubiquitin protein ligase E3A” OR “Ubiquitin-protein ligase E3A” OR “HECT-type ubiquitin transferase E3A” OR “Oncogenic protein-associated protein E6-AP”) OR (Pten OR “Phosphohydrolase” OR “PTEN Phosphohydrolase”[MeSH] OR “Mmac1” OR “Mutated in multiple advanced cancers 1” OR “Phosphatase and tensin homolog” OR “phosphatidylinositol 3,4,5-trisphosphate 3-phosphatase” OR “dual-specificity protein phosphatase”) OR (Nlgn3 OR “neuroligin-3” OR “neuroligin 3” OR “gliotactin homolog”) OR (Shank3 OR Shank3b OR “SH3-ankyrin domain gene 3 protein” OR “SH3 and multiple ankyrin repeat domains protein 3” OR Kiaa1650 OR Prosap2 OR “Proline-rich synapse-associated protein 2” OR ProSAP2 OR “SPANK-2”) OR (Mecp2 OR “Methyl-CpG-Binding Protein 2”[MeSH] OR “Methyl-CpG-binding protein 2” OR “MeCp-2”) OR (Fmr1 OR “Synaptic functional regulator FMR1” OR “Fragile X mental retardation protein 1 homolog” OR “FMRP” OR “mFmr1p” OR “Fmr-1”) OR (“BTBR T+Itpr3tf/J” OR “BTBR” OR “Itpr3tf/J”)) OR ((autis* OR neonates OR prenatal OR postnatal OR perinatal OR pups OR offspring OR gestational OR weaning) AND (“LPS” OR Lipopolysaccharide OR “VPA” OR “Valproic Acid”[MeSH] OR “valproate” OR valproic OR “polyinosinic:polycytidylic acid” OR “Poly I-C”[MeSH] OR “Poly I:C” OR “maternal immune activation” OR “immune activation”))) AND (“Models, Animal”[MeSH] OR “Animal Experimentation”[MeSH] OR “Disease Models, Animal”[MeSH] OR rodent* OR “Murinae”[MeSH] OR “murinae” OR “Mice”[MeSH] OR “Rats”[MeSH] OR “animal model” OR “animal models” OR rat OR rats OR mouse OR mice OR “Rattus” OR “Mus”)

Database/Platform	Web of Science/Clarivate Analytics
Limits	in: “all fields”<br>date range: all years
Search date and number of hits	05/nov/2020 09:21 am - 14,202
Search query	(((Ube3a OR “ubiquitin protein ligase E3A” OR “Ubiquitin-protein ligase E3A” OR “HECT-type ubiquitin transferase E3A” OR “Oncogenic protein-associated protein E6-AP”) OR (Pten OR “Phosphohydrolase” OR “PTEN Phosphohydrolase” OR “Phosphatase and Tensin Homologue on Chromosome Ten Protein” OR “Mutated In Multiple Advanced Cancers 1 Protein” OR “Mmac1” OR “Mutated in multiple advanced cancers 1” OR “Phosphatase and tensin homolog” OR “phosphatidylinositol 3,4,5-trisphosphate 3-phosphatase” OR “dual-specificity protein phosphatase”) OR (Nlgn3 OR “neuroligin-3” OR “neuroligin 3” OR “gliotactin homolog”) OR (Shank3 OR Shank3b OR “SH3-ankyrin domain gene 3 protein” OR “SH3 and multiple ankyrin repeat domains protein 3” OR Kiaa1650 OR Prosap2 OR “Proline-rich synapse-associated protein 2” OR ProSAP2 OR “SPANK-2”) OR (Mecp2 OR “Methyl-CpG-Binding Protein 2” OR “MeCp-2” OR “Methyl CpG Binding Protein 2”) OR (Fmr1 OR “Synaptic functional regulator FMR1” OR “Fragile X mental retardation protein 1 homolog” OR “FMRP” OR “mFmr1p” OR “Fmr-1”) OR (“BTBR T+Itpr3tf/J” OR “BTBR” OR “Itpr3tf/J”)) OR ((autis* OR neonates OR prenatal OR postnatal OR perinatal OR pups OR offspring OR gestational OR weaning) AND ((“LPS” OR Lipopolysaccharide) OR (“VPA” OR “Valproic Acid” OR “2-Propylpentanoic Acid” OR “Divalproex” OR “Depakene” OR “Convulsofin” OR “Depakote” OR “Dipropyl Acetate” OR “Divalproex Sodium” OR “Vupral” OR “Propylisopropylacetic Acid” OR “Ergenyl” OR “Depakine” OR “valproate” OR valproic) OR (“polyinosinic:polycytidylic acid” OR “Poly I-C” OR “Polyriboinosinic-Polyribocytidylic Acid” OR “Polyriboinosinic Polyribocytidylic Acid” OR “Polyribose Inosin-Cytidil” OR “Polyribose Inosin Cytidil” OR “Polyinosinic-Polycytidylic Acid” OR “Polyinosinic Polycytidylic Acid” OR “Poly(rI).Poly(rC)” OR “Poly I:C” OR “maternal immune activation” OR “immune activation”)))) AND (“Animal Experimentation” OR “animal disease model” OR “animal disease models” OR rodent* OR “Murinae” OR “Rats” OR “animal model” OR “animal models” OR rat OR rats OR “Rattus norvegicus” OR “Rattus” OR mouse OR mice OR “Mus musculus” OR “Mus” OR “experimental animal” OR “laboratory animal” OR “laboratory animals” OR “animal research” OR “animal experiment”)

Database/Platform	Scopus/Elsevier
Limits	in: “Article title, Abstract, Keywords”<br>date range: all years
Search date and number of hits	05/nov/2020 09:25 am - 18,336
Search query	TITLE-ABS-KEY((((Ube3a OR “ubiquitin protein ligase E3A” OR “Ubiquitin-protein ligase E3A” OR “HECT-type ubiquitin transferase E3A” OR “Oncogenic protein-associated protein E6-AP”) OR (Pten OR “Phosphohydrolase” OR “PTEN Phosphohydrolase” OR “Phosphatase and Tensin Homologue on Chromosome Ten Protein” OR “Mutated In Multiple Advanced Cancers 1 Protein” OR “Mmac1” OR “Mutated in multiple advanced cancers 1” OR “Phosphatase and tensin homolog” OR “phosphatidylinositol 3,4,5-trisphosphate 3-phosphatase” OR “dual-specificity protein phosphatase”) OR (Nlgn3 OR “neuroligin-3” OR “neuroligin 3” OR “gliotactin homolog”) OR (Shank3 OR Shank3b OR “SH3-ankyrin domain gene 3 protein” OR “SH3 and multiple ankyrin repeat domains protein 3” OR Kiaa1650 OR Prosap2 OR “Proline-rich synapse-associated protein 2” OR ProSAP2 OR “SPANK-2”) OR (Mecp2 OR “Methyl-CpG-Binding Protein 2” OR “MeCp-2” OR “Methyl CpG Binding Protein 2”) OR (Fmr1 OR “Synaptic functional regulator FMR1” OR “Fragile X mental retardation protein 1 homolog” OR “FMRP” OR “mFmr1p” OR “Fmr-1”) OR (“BTBR T+Itpr3tf/J” OR “BTBR” OR “Itpr3tf/J”)) OR ((autis* OR neonates OR prenatal OR postnatal OR perinatal OR pups OR offspring OR gestational OR weaning) AND ((“LPS” OR Lipopolysaccharide) OR (“VPA” OR “Valproic Acid” OR “2-Propylpentanoic Acid” OR “Divalproex” OR “Depakene” OR “Convulsofin” OR “Depakote” OR “Dipropyl Acetate” OR “Divalproex Sodium” OR “Vupral” OR “Propylisopropylacetic Acid” OR “Ergenyl” OR “Depakine” OR “valproate” OR valproic) OR (“polyinosinic:polycytidylic acid” OR “Poly I-C” OR “Polyriboinosinic-Polyribocytidylic Acid” OR “Polyriboinosinic Polyribocytidylic Acid” OR “Polyribose Inosin-Cytidil” OR “Polyribose Inosin Cytidil” OR “Polyinosinic-Polycytidylic Acid” OR “Polyinosinic Polycytidylic Acid” OR “Poly(rI).Poly(rC)” OR “Poly I:C” OR “maternal immune activation” OR “immune activation”))))) AND TITLE-ABS-KEY((“Animal Experimentation” OR “animal disease model” OR “animal disease models” OR rodent* OR “Murinae” OR “Rats” OR “animal model” OR “animal models” OR rat OR rats OR “Rattus norvegicus” OR “Rattus” OR mouse OR mice OR “Mus musculus” OR “Mus” OR “experimental animal” OR “laboratory animal” OR “laboratory animals” OR “animal research” OR “animal experiment”))
