# Supplementary material for: Behavioral manifestations in rodent models of autism spectrum disorder: protocol for a systematic review and network meta-analysis
Source: Syst Rev. 2022 Jul 26;11:150. doi: 10.1186/s13643-022-02028-w (PMC9327140; doi:10.1186/s13643-022-02028-w)
Supplement: Supplementary file 2 — Additional file 2. Behavioral outcomes. List of behavioral categories and examples of experimental paradigms. [file 13643_2022_2028_MOESM2_ESM.pdf]

SOCIAL BEHAVIOUR		
**Pheno Term (PhenoID)**	**Pheno Definition**	**Experimental Paradigm**
Aggression (AM1600000)	Domineering, assaultive or forceful physical action	Reciprocal social interaction test; Resident-intruder test; Competitive courtship assay; Mirror test; Home cage behavior; General observations; Neurological exam battery; Three-chamber social approach test; Open field test
Allogrooming (AM1600001)	Behavior in which individuals in a cohort clean or maintain one another's outer body or appearance	General observations; Reciprocal social interaction test; Visible burrow system; Open field test; Home cage behavior
Huddling Behavior (AM1600002)	Tendency of mice to pile together when sleeping	Home cage behavior; Visible burrow system; Novel cage test; General observations
Inanimate object preference (AM1600003)	Preference or curiosity towards inanimate object and play over a social stimulus	Novel object interaction test; Novel object recognition test; Reciprocal social interaction test; Novel object exploration test; Three-chamber social approach test; Open field test
Juvenile play (AM1600004)	Social behavior at a young age towards another juvenile social stimulus mouse, including sniffing, crawling and other play behavior	Reciprocal social interaction test; Three-chamber social approach test; Open field test; Home cage behavior; General observations
Mating behavior (AM1600005)	Initiation of sexual behavior	Competitive courtship assay; General observations; Scent marking test; Courtship conditioning assay; Reciprocal social interaction test; Open field test; Mating behavior test
Social dishabituation (AM1600006)	Temporary recovery of response to a stimulus when a novel social stimulus is presented	Reciprocal social interaction test; Social recognition test; Habituation-dishabituation test
Nest building behavior (AM1600007)	Behavior of building sleeping nests out of any available materials	Nest building assay; Home cage behavior; General observations; Open field test
Passive social behavior (AM1600008)	Social interaction of stimulus mouse towards experimental mouse	Reciprocal social interaction test
Rearing behavior (AM1600009)	Ability or desire to stand vertically on hind limbs or social behavior	Object-place recognition test; Three-chamber social approach test; Home cage behavior; Observation of repetitive behavior; Reciprocal social interaction test; Open field test; Resident-intruder test; Ambulation recordings; Barnes maze test; Elevated plus maze test; General observations; Novel cage test

Self grooming: social contexto (AM1600010)	Behavior of cleaning or keeping outward self appearance tidy in the presence of social stimuli	Reciprocal social interaction test; Three-chamber social approach test; Grooming behavior assessments; General observations
Social habituation (AM1600011)	Progressive decline of behavioral response probability with an exposure to a novel social stimulus, such as a conspecific	General observations; Habituation-dishabituation test; Social recognition test; Olfactory habituation-dishabituation test; Reciprocal social interaction test; Resident-intruder test
Social approach (AM1600012)	Preference for a social target, such as an unfamiliar stimulus mouse, over a non-social target, such as an inanimate object, or empty environment	Three-chamber social approach test; Open field test; Reciprocal social interaction test; Partition test; Social recognition test; Resident-intruder test; Olfactory discrimination test
Social dominance (AM1600013)	Exhibition of domineering, assaultive posture or hostile physical action toward other mice during instigated confrontations	Tube test of social dominance; Dominance hierarchy formation
Social interaction (AM1600014)	Normal behavior towards social targets, measured by time spent in direct contact with stimulus mice	Resident-intruder test; Reciprocal social interaction test; Three-chamber social approach test; Open field test; Social transmission of food preference; Social recognition test; Elevated plus maze test; Light-dark exploration test; Y-maze test; Partition test; Hole-board test; Running wheel test; Novel cage test; Home cage behavior; Interaction with caged adult; Modified open field test with novel object; T-maze test; Partition test in tank; Novel object exploration test; General observations; Separation-reunion test; Novel object interaction test; Elevated T-maze test; Dominance hierarchy formation; Visible burrow system; Radial maze test
Social cohesion (AM1600015)	Preference of an organism to maintain a minimum or maximum distance from conspecifics.	Social space assay
Social interaction: opposite sex (AM1600016)	Interaction of male mice with female stimulus mice, to initiate mating behavior	Spectographic analysis of vocalization recordings; Reciprocal social interaction test; Three-chamber social approach test; Scent stimulus
Social interaction: with juveniles (AM1600017)	Interaction of adult mice with juveniles (aged 2-3 weeks)	Reciprocal social interaction test; Open field test
Social memory (AM1600018)	Preference to interact with a novel stimulus mouse rather than a familiar mouse	Three-chamber social approach test; Social recognition test; Partition test; General observations; Reciprocal social interaction test; Courtship conditioning assay; Olfactory habituation-

		dishabituation test; Courtship assay; Open field test; Resident-intruder test
Social place preference (AM1600019)	Conditioned preference for objects (like bedding material) shared between cage mates - from social cues	Olfactory discrimination test; Social conditioned place preference
Social scent marking or recognition (AM1600020)	Preference for social odor, such as urine or used bedding, over a non-social odor and recognition of unfamiliar urine, maternal scent	Olfactory discrimination test; Olfactory habituation-dishabituation test; Homing test; Three-chamber social approach test; Reciprocal social interaction test; Hole-board test; Western blot; General observations; Scent marking test; Maternal scent recognition test
Social transmission of food preference (AM1600021)	Finding location of buried food following other mice	Social transmission of food preference; Reciprocal social interaction test
Social withdrawal (AM1600022)	Tendency to refrain from participating in social situations and to seek isolation	Three-chamber social approach test; Visible burrow system; Reciprocal social interaction test; Open field test; Shoaling assay; Home cage behavior
Nest utilization behavior (AM1600023)	Behavior of utilizing nests for resting/sleeping	Nest utilization assay
Social memory: short-term social memory (AM1600024)	Preference to interact with a novel stimulus conspecific rather than a familiar conspecific, tested after a short interval, e.g. 5 minutes	Reciprocal social interaction test; Courtship assay
Social memory: long-term social memory (AM1600025)	Preference to interact with a novel stimulus conspecific rather than a familiar conspecific, tested after a long interval, e.g. 24 hours	Courtship assay; Reciprocal social interaction test
Social fear conditioning: learning (AM1600026)	Acquisition of affective behavior based on social cues produced by a conspecific, which can imbue associated environmental cues and contexts with affective significance; based on conspecific exposure to fear conditioning paradigm where a conditioned stimulus is associated with an unconditioned stimulus	Fear conditioning test
Social fear conditioning: memory of cue (AM1600027)	Association of environmental cue (e.g. tone) to affective distress; based on conspecific exposure to	Fear conditioning test

	fear conditioning paradigm where a conditioned stimulus is associated with an unconditioned stimulus	
Social fear conditioning: memory of contexto (AM1600028)	Association of environmental context to affective distress; based on conspecific exposure to fear conditioning paradigm where a conditioned stimulus of a conspecific introduced in a wired cage within the test chamber, is associated with an unconditioned stimulus of foot shocks	Fear conditioning test
Mating behavior: Male courtship behavior (AM1600029)		Mating behavior test; General observations; Courtship assay
Mating behavior: Male courtship behavior: prior orientation (AM1600030)		General observations; Mating behavior test
Mating behavior: Male courtship behavior: wing extension (AM1600031)		General observations; Mating behavior test
Mating behavior: Male courtship behavior: proboscis-mediated licking (AM1600032)		Mating behavior test; General observations
Social cohesion: Shoaling behavior (AM1600033)	Fish behavior where a group of fishes swim together for social reasons, to find food, or combat predators.	Three-chamber social approach test; Shoaling assay
Mating behavior: Male courtship behavior: audible vocalization (AM1600034)	The process during wing vibration where the male insect produces a species-specific acoustic signal as part of courtship behavior called a love song.	Sound Recordings
Mating behavior: Male courtship behavior: chaining (AM1600035)	The formation of chains of multiple males following each other during courtship.	General observations
Aggression: auditory stimulus induced (AM1600036)	Aggressive behavior induced by an auditory stimulus and based on competition between males of the same species over access to resources such as females,	Competitive Courtship Assay

	dominance, status, etc. and characterized by noise, threats, and is often less injurious.	
Social cohesion: kin preference (AM1600037)	Preference for related individuals over unrelated individuals of the same species	Shoaling assay
Empathy (AM1600038)	Ability to understand the experience or emotion of another, usually in distress	
**REPETITIVE BEHAVIOUR**		
**Pheno Term (PhenoID)**	**Pheno Definition**	**Experimental Paradigm**
Allogrooming: perseveration (AM1300000)	Excessive allogrooming, a behavior in which individuals in a cohort clean or maintain one another's outer body or appearance	Grooming behavior assessments; Observation of repetitive behavior; Resident-intruder test; Home cage behavior
Circling (AM1300001)	Repeated, compulsive movement in a circle, often associated with inner ear defects	Spontaneous movement analysis; Open field test; Home cage behavior; General observations; Larval foraging assay; Novel cage test; Three-chamber social approach test
Head bobbing (AM1300002)	Compulsive up and down movement of the head	Hole-board test; Novel cage test; General observations; Elevated plus maze test
Self injurious behavior (AM1300003)	Compulsive behavior that results in self harm	General observations; Open field test
Head shaking (AM1300004)	Compulsive movement of the head in the horizontal plane	General observations
Head tossing (AM1300005)	Compulsive flailing of the head in multiple directions	Spontaneous movement analysis
	Continuing repetitive or compulsive behavior that may arise due to lack of habituation to an environment or object or mouse, despite adequate exposure	Y-maze test; Barnes maze test; Three-chamber social approach test; Morris water maze test; Eight-arm radial maze test; T-maze test; Spatial discrimination assay; Accelerating rotarod test; Repetitive novel object interaction test; Novel object interaction test; Observation of repetitive behavior; Marble-burying test; Running wheel test; Nest building assay
Perseveration (AM1300006)	A repetitive behavior trying to unearth objects from cage bedding or burying marbles	Marble-burying test; Home cage behavior; Burrowing test; Resident-intruder test; Reciprocal social interaction test;

		Observation of repetitive behavior; Three-chamber social approach test; Object-place recognition test
Repetitive nose pokes (AM1300008)	Repetitive pokes on the same hole in the holeboard nose poke assay	Hole-board test; Spontaneous movement analysis
Self grooming: perseveration (AM1300009)	Behavior of cleaning or keeping outward self appearance tidy in a compulsive manner	General observations; Grooming behavior assessments; Open field test; Home cage behavior; Reciprocal social interaction test; Novel cage test; Induced scratching response; Observation of repetitive behavior; Novel object recognition test; Three-chamber social approach test; Splash test to evoke grooming behavior; Visible burrow system; Resident-intruder test
Self Scratching (AM1300010)	Compulsive scraping of the skin, usually with the nails, and increased grooming	Grooming behavior assessments
Stereotypy (AM1300012)	Repetitive, invariant, persistent motor patterns that do not appear to be purposeful and cannot be categorized as any of the other repetitive behaviors	Marble-burying test; Observation of repetitive behavior; Home cage behavior; Ambulation recordings; Open field test; General observations; Reciprocal social interaction test; Repetitive novel object interaction test; Force plate actometer test; T-maze test
Stereotypy: climbing (AM1300013)	Stereotypic climbing on the ceiling of the cage for no observable reason or function	Home cage behavior
Vertical jumping or back flipping (AM1300014)	Continous bouts of jumping or back flipping without provocation or stimuli	Open field test; Home cage behavior; Observation of repetitive behavior; Grooming behavior assessments; General observations; Modified open field test with novel object
Stereotypy: chewing behavior (AM1300015)	Repetitive or persistent chewing behavior of an object, e.g. a wooden block	General observations; Observation of repetitive behavior
**COMMUNICATIONS**		
**Pheno Term (PhenoID)**	**Pheno Definition**	**Experimental Paradigm**
Audible vocalization (AM200000)	Production of vocal audible sound	Monitoring vocalizations; Monitoring ultrasonic vocalizations; Resident-intruder test; General observations
Ultrasonic vocalization (AM200001)	Production of vocal ultrasonic sound; production of vocal ultrasonic sound in response to a variety of environmental triggers, including separation from	Monitoring ultrasonic vocalizations; Reciprocal social interaction test

	mother, interaction with other strange or familiar conspecifics of same or opposite sex	
Ultrasonic vocalization: Interaction induced (AM200002)	Ultrasonic vocalization induced in juvenile (2-4 weeks) or adult mice during social interaction with stimulus mice	Monitoring ultrasonic vocalizations; Reciprocal social interaction test; Spectographic analysis of vocalization recordings
Ultrasonic vocalization: Interaction induced: opposite sex stimulus (AM200003)	Ultrasonic vocalization induced in juvenile (2-4 weeks) or adult during social interaction with stimulus mice of the opposite sex	Monitoring ultrasonic vocalizations; Reciprocal social interaction test; Scent marking test; Song analysis
Ultrasonic vocalization: Isolation induced (AM200004)	Ultrasonic vocalization induced in young pups following separation from mother	Monitoring ultrasonic vocalizations; Spectographic analysis of vocalization recordings; Reciprocal social interaction test
Ultrasonic vocalization: Prosocial (AM200005)	Generation and rate of pro-social or hedonic or pleasurable ultrasonic vocalizations (USVs) characterized by a frequency of 50 kHz emitted in pleasurable, enjoyable, appetitive, and rewarding situations	Monitoring ultrasonic vocalizations
Ultrasonic vocalization: Alarm (AM200006)	Generation and rate of alarm or aversive ultrasonic vocalizations (USVs) characterized by a frequency of 22 kHz, emitted in dangerous and threatening situations, or situations causing discomfort, frustration, and significant stress and anxiety	Monitoring ultrasonic vocalizations
Ultrasonic vocalization: Monotonous (AM200007)	Generation and rate of non-frequency modulated ultrasonic vocalizations (USVs) characterized by a limited bandwidth, usually less than 7 kHz	Monitoring ultrasonic vocalizations
Ultrasonic vocalization: Interaction induced: opposite sex stimulus: simple syllables (AM200008)	Ultrasonic vocalization of syllables that are included in longer series of vocalizations called sequences (that are combined together to produce a 'song'), produced without any pitch jumps, hence simple in composition, in response to opposite sex stimulus	Song analysis
Ultrasonic vocalization: Interaction induced: opposite sex stimulus: complex syllables (AM200009)	Ultrasonic vocalization of syllables that are included in longer series of vocalizations called sequences (that are combined together to produce a 'song') containing	Song analysis

	one or more, downward or upward, pitch jump: hence complex, in response to an opposite sex stimulus	
Ultrasonic vocalization: Interaction induced: same sex stimulus (AM200010)	Ultrasonic vocalization of syllable sequences or song in response to same sex stimulus	Song analysis; Reciprocal social interaction test; Monitoring ultrasonic vocalizations
**EMOTION**		
**Pheno Term (PhenoID)**	**Pheno Definition**	**Experimental Paradigm**
Acute Stress response (AM400000)	Reaction of the sympathetic nervous system, including an increase in core body temperature, in response to an acute stressful event	Persecution with pen net; Ambulatory activity after saline injection; Novel cage test; Forced swim test; Fear conditioning test; Restraint test; Immunoassay; Tail suspension test; Acute social isolation; In situ hybridization (ISH); Open field test; Fecal pellet count
Anxiety (AM400001)	Anxiety-like behavior in rodents where the emotional response, likened to human anxiety, is elicited by aversive stimuli that are diffuse, unpredictable, distal and/or of long duration. These include avoidance and defensive behaviors.	Open field test; Elevated plus maze test; Morris water maze test; Light-dark exploration test; Marble-burying test; Elevated zero maze test; Novelty-suppressed feeding paradigm; Reciprocal social interaction test; Novel object exploration test; Successive alley test; Modified open field test with novel object; Defensive burying test; Measurement of stress-induced hyperthermia; Three-chamber social approach test; Novel cage test; Novel tank diving test; General observations; Restraint-stress induced corticosterone levels; Bottom dwelling test; Zero maze test; T-maze test; Mouse defense test battery
Depression (AM400003)	Defeated emotional state in response to a stressful situation or stimulus, where the trigger is perceived defeat in a hierarchical struggle for resources, mating choices or entrapment/despair. These can be measured in rodents by reduced psychomotor activity (immobility), alterations in appetite (anhedonia) and sleep patterns.	Forced swim test; Tail suspension test; Sucrose preference test; Modified forced swim test; Measurement of emotion

Exploratory activity (AM400004)	Duration or frequency of investigation of an object or place, usually increased for unfamiliar or object/space or if there is a gap in reintroduction	Three-chamber social approach test; Novel object recognition test; Open field test; Home cage behavior; Hole-board test; Textured novel object recognition test (T-NORT); Object-place recognition test; Elevated plus maze test; Marble-burying test; General observations; Novel object interaction test; Object preference test; Novel cage test; Novel object exploration test; Resident-intruder test; Reciprocal social interaction test; Y-maze test; Light-dark exploration test; Radial maze test; Puzzle box test; Operant conditioning paradigm
Exploratory activity: Habituation (AM400005)	Habituation to a novel environment, typically measured by a decrease of exploratory activity after exposure of a few minutes. This is non-associative learning.	Open field test; Barnes maze test; Three-chamber social approach test; Novel object recognition test; Home cage behavior; Habituation-dishabituation test
Fear response (AM400006)	Emotional response related to anticipation of specific pain or danger	Startle response test; Defensive withdrawal test; Open field test; Novel object exploration test; Elevated T-maze test; Light-dark exploration test; Fear conditioning test; Mouse defense test battery; Response to olfactory stimuli
Response to novelty (AM400007)	Amount of exploration of a novel object, situation or environment	Open field test; Eight-arm radial maze test; Novel object interaction test; Object-place recognition test; Novel object recognition test; Novel cage test; Elevated T-maze test; Light-dark exploration test; Elevated plus maze test; Habituation-dishabituation test; Annex test; Zero maze test
Thigmotaxis (AM400008)	Preference for staying at the perimeter walls of an open area, which may be an indicator of anxiety	Open field test; Morris water maze test; Gentle touch test
Habituation to aversive stimuli (AM400009)	Decrease in startle /reflex amplitude following several exposures to aversive stimuli like acoustic startle, as habituation occurs to the stimulus following continued or prolonged conditioning. This is non-associative learning.	Acoustic startle reflex test; Light-off jump reflex habituation assay; Muscle reflex; Light-off startle jump
Fear response: predator stimulus (AM400010)	Response to the presence of predator (which can be sensed through scent, computer generated graphics or videos on sides of tanks), expressed in most organisms	

	as some form of motor response like bottom dwelling in fish, or passive avoidance in rodents	
**LEARNING AND MEMORY**		
**Pheno Term (PhenoID)**	**Pheno Definition**	**Experimental Paradigm**
Cognitive flexibility (AM700000)	Ability to restructure previously learned concepts with new information to manage new situations, for example, reversal learning	Water T-maze test; Morris water maze test; Radial maze test; Spatial discrimination assay; Conflict place avoidance test; Barnes maze test; Operant conditioning paradigm; T-maze test; Fear conditioning test; Two-choice digging test; Water Y-maze; Puzzle box test; Running wheel test; Clock-maze test
Cognitive flexibility: Associative learning (AM700001)	Ability to restructure previously learned concepts with new information to manage new situations; in relation to the acquisition of associative memory, which establishes a relationship between two stimuli, or between a stimulus and a behavioral response	Conditioned place preference test; Fear conditioning test; Set-shifting task; Hole-board test; T-maze test; Morris water maze test
Conditioned taste aversion (AM700002)	Classical conditioned avoidance of certain foods (CS) following a previous experience of malaise	Conditioned taste aversion test
Cued or contextual fear conditioning (AM700003)	Storage of information that establishes an association between an aversive experience (the unconditioned stimulus (US), usually a shock) and a neutral stimulus (the conditioned stimulus (CS), usually an auditory cue, or a neutral environment)	Fear conditioning test; Forced swim test; Passive avoidance test; Active avoidance test
Cued or contextual fear conditioning: Context discrimination (AM700004)	Ability to discriminate a novel environment from a familiar environment (the conditioned stimulus or CS) where there was a learned association with an aversive experience (the unconditioned stimulus or US, usually a shock)	Fear conditioning test
Cued or contextual fear conditioning: Memory of contexto (AM700005)	Storage of information that establishes an association, lasting days, between an aversive experience (the unconditioned stimulus or US, usually a shock) and a neutral, unchanging environment (the conditioned stimulus or CS)	Fear conditioning test; Open field test; Novel cage test; Foot shock test

Cued or contextual fear conditioning: Memory of context: Long term recall (AM700006)	Ability to establish a remote memory, lasting weeks, of an association between an aversive experience (the unconditioned stimulus or US, usually a shock) and a neutral, unchanging environment (the conditioned stimulus or CS)	Fear conditioning test
Cued or contextual fear conditioning: Memory of cue (AM700007)	Storage of information that establishes an association, lasting days, between an aversive experience (the unconditioned stimulus or US, usually a shock) and a neutral auditory cue (the conditioned stimulus or CS)	Fear conditioning test; Operant self-learning paradigm; Passive avoidance test; Active avoidance test; Conditioned place preference test
Cued or contextual fear conditioning: Passive avoidance (AM700008)	Storage of information that establishes a relationship between the environment and an unpleasant or punishing stimulus, such as a shock, applied with a prolonged latency	Passive avoidance test; Open field test
Cued or contextual fear conditioning: Trace fear conditioning (AM700009)	Storage of information that establishes an association between an aversive experience (the unconditioned stimulus or US, usually a shock) and a neutral auditory cue (the conditioned stimulus or CS) where there is an empty interval (trace) that separates the cessation of the CS from the onset of the US	Fear conditioning test
Cued or contextual fear conditioning: Latent inhibition (AM700010)	Impairment in a conditioned response seen after repeated unpaired presentations of the conditioned stimulus (CS) prior to pairing it with the unconditioned stimulus (US)	Fear conditioning test
Eye blink conditioning (AM700011)	Ability to learn to blink in anticipation of an aversive stimulus (e.g., an air puff to the eyelid) following repeated pairings with a neutral stimulus (e.g., a tone), at a precise timing between the conditioned and unconditioned stimuli	Eyeblink conditioning
Object recognition memory (AM700012)	Ability to recognize objects previously encountered, usually measured by decreased exploration of familiar objects	Novel object recognition test; Running wheel test; General observations; Object-place recognition test; Open field test; Visual lateralization novel object laterization (VLNOR); Grooming

		behavior assessments; Reciprocal social interaction test; Novel object exploration test
Olfactory learning and memory (AM700013)	Ability to exhibit a differential response to olfactory stimuli that is achieved by the reinforcement of the desired response for each particular olfactory stimulus	Olfactory habituation-dishabituation test; Hole-board test; Fear conditioning test; Appetitive odor preference test; Operant conditioning paradigm
Procedural learning (AM700014)	Acquisition of implicit memory, that which does not require conscious attention for recall, for example, habits, perceptual or motor strategies, associative and non-associative conditioning	Active avoidance test; Running wheel test; Accelerating rotarod test
Cued or contextual fear conditioning: Memory of cue: Long term recall (AM700015)	Ability to establish a remote memory, lasting weeks, of an association between an aversive experience (the unconditioned stimulus or US, usually a shock) and a neutral auditory cue (the conditioned stimulus or CS)	Fear conditioning test
Reward reinforced choice behavior (AM700016)	Learned behavior of making a choice that has a higher probability of increased reward	T-maze test; Y-maze test; Operant conditioning paradigm; Biological motion perception test; Conditioned place preference test; Five-choice serial reaction time test (5-CSRTT); Two-choice digging test; Delayed non-match to sample T-maze test; Barnes maze test; Sucrose preference test
Spatial learning (AM700017)	Ability to ascertain or acquire spatial location information in order to improve navigation or other behavior using such location cues	Barnes maze test; Morris water maze test; Object-place recognition test; T-maze test; Radial maze test; Water T-maze test; Water Y-maze; Clock-maze test; Labyrinth maze test; Spatial discrimination assay; Operant self-learning paradigm
Spatial reference memory (AM700018)	Ability to recall spatial location information from previous encounters or training sessions in order to navigate or perform other behavior using such location cues	Morris water maze test; T-maze test; Barnes maze test; Object-place recognition test; Radial maze test; Water Y-maze; Open field test; Passive avoidance test; Y-maze test; Plus-shaped water maze test
Spatial working memory (AM700019)	Ability to spontaneously process spatial location information in order to navigate or perform other behavior using such location cues, without previous encounters or training at that location	Morris water maze test; Radial maze test; Water Y-maze test; Y-maze test; T-maze test; Barnes maze test; Delayed non-match to place test; Water T-maze test; Nose poke test; Eight-arm radial maze test; Object-place recognition test
Swim distance (AM700020)	Total distance swum by the test mouse, usually in the Morris water maze	

Eye blink conditioning: extinction and reactivation (AM700021)	Ability to extinguish or forget a previously learned eye blink conditioned response (CR) after being exposed to conditioned stimulus (CS) without unconditioned stimulus (US), sometimes followed by reactivation or relearning of the CR to the same or modified CS	Eyeblink conditioning
Stimulus-response learning (AM700022)	Ability to learn to perform a particular behavior when a certain stimulus is present, for example using a proximal visual cue to complete a task, as opposed to distal contextual cues	Barnes maze test; Sucrose preference test
Episodic-like memory (AM700023)	Temporal and spatial recall required for tasks with long delay	Morris water maze test; Radial maze test; Puzzle box test; Object-place recognition test
Cued or contextual fear conditioning: Extinction (AM700024)	Ability to extinguish aversive memories associated with a traumatic incident	Fear conditioning test
Cognitive flexibility: distractor suppression (AM700025)	Differentiating distracting stimuli from relevant stimuli during training to learn a task, similar to attention tasks in people	Operant conditioning paradigm
Object recognition memory: long-term recall (AM700026)	Ability to recognize objects previously encountered after being exposed to a familiar and a novel object following an longer interval (dependent on experimental designa nd authors), usually measured by decreased exploration of familiar objects	Visual lateralization novel object laterization (VLNOR); Novel object recognition test
Cued or contextual fear conditioning: Active avoidance (AM700027)	Fear-motivated storage of information that establishes an association between an aversive unconditional stimulus and specific cues (conditional stimulus) in a variable environment	Active avoidance test
Cued or contextual fear conditioning: Memory of context: Short-term memory (AM700028)	Storage of information that establishes a short-term association, lasting minutes or hours, between an aversive experience (the unconditioned stimulus or US, usually a shock) and a neutral, unchanging environment (the conditioned stimulus or CS)	Fear conditioning test

Cued or contextual fear conditioning: Memory of cue: Short-term memory (AM700029)	Storage of information that establishes a short-term association, lasting minutes or hours, between an aversive experience (the unconditioned stimulus or US, usually a shock) and a neutral auditory cue (the conditioned stimulus or CS)	Fear conditioning test
Reward reinforced choice behavior: learning (AM700030)		T-maze test
Reward reinforced choice behavior: short-term memory (AM700031)		T-maze test
Reward reinforced choice behavior: long-term memory (AM700032)		T-maze test
Memory: short-term memory (AM700033)	The memory process that deals with the storage, retrieval and modification of information received a short time (up to about 30 minutes) ago. This type of memory is typically dependent on direct, transient effects of second messenger activation.	
Memory: medium-term memory (AM700034)	The memory process that deals with the storage, retrieval and modification of information received at a time ago that is intermediate between that of short and long term memory (30min - 7hrs in Drosophila melanogaster).	
Cognitive flexibility: Associative learning: operant self-learning (AM700035)		Operant self-learning paradigm
**SENSORY**		
**Pheno Term (PhenoID)**	**Pheno Definition**	**Experimental Paradigm**
Startle response: acoustic stimulus (AM1500000)	Threshold for reflex response to auditory stimuli, usually measured by amplitude of whole body flinch	Acoustic startle reflex test; Prepulse inhibition; Preyer's Reflex test; Foot shock test; Startle response test; Neurological exam battery; General observations

Ear twitch reflex or Preyer's reflex (AM1500002)	Response to an auditory stimulus by a characteristic ear twitch	Negative geotaxis test; Grasping reflex test; Righting reflex test; Preyer's Reflex test
Foot shock sensitivity (AM1500003)	Level of perception of pain inflicted by an electric shock to the feet, specifically to check for pain threshold levels before fear conditioning or related paradigms.	Fear conditioning test; Foot shock test; Passive avoidance test
Head twitch response (AM1500004)	Repetitive movement of the head in the horizontal plane	Head twitch response
Hearing (AM1500005)	Function or activity of the auditory system or any of its parts	Auditory nerve recordings; Auditory test; Auditory brainstem response test; Startle response test; Whole-cell patch clamp; Preyer's Reflex test; Acoustic startle reflex test; Prepulse inhibition
Itch sensation (AM1500008)	Scratching behavior in response to itch stimulus	Intrathecal injection; Intradermal injection
Olfaction (AM1500011)	Function or activity of the olfactory system or any of its parts	Olfactory discrimination test; Neurological exam battery; Predator odor (TMT) test; Buried food test; Home cage odor preference test; Response to olfactory stimuli; Olfactory habituation-dishabituation test; Urine preference test; Olfactory trap; Scent marking test; Three-chamber social approach test; Hole-board test; Olfactory investigation test; Novel object exploration test; General observations
Pain or nociception (AM1500012)	Ability to sense pain at a certain threshold	Hot plate test; Von Frey filament test; Paw injection test; Foot shock test; Tail flick test; Swim stress test; Paw withdrawal test; Intraperitoneal injection; Tail pinch test; Fear conditioning test; Aversive drinking test
Sensorimotor gating (AM1500014)	Process by which inhibitory neural pathways filter multiple stimuli and allow attention to be focused on one stimulus, usually measured by pre-pulse inhibition (PPI)	Prepulse inhibition; Adhesive tape test; Acoustic startle reflex test; Startle response test; Electromyography (EMG)
Taste (AM1500015)	Perception of taste	Sucrose preference test

Touch<br>(AM1500016)	Ability to sense contact with object in response to tactile stimuli	Disc assay; Von Frey filament test; Gentle touch test; Response to air puff; General observations; Foot shock test; Adhesive tape test; Hot plate test; Tail flick test
Vestibulo-ocular reflex<br>(AM1500017)	Nystagmus or deviation of the eyes in response to stimulation of the vestibular system in which impulses are conveyed from the semicircular canals and the otolithic membrane to the oculomotor nerve	Swim test; Righting reflex test
Vision<br>(AM1500018)	Function or activity of the ocular system or any of its parts	Morris water maze test; Forepaw reaching test; Optomotor response to pattern illuminance; Arena test with vertical stripe; Disc assay; General observations; Ophthalmological evaluation; Y-maze test; Neurological exam battery; Cliff avoidance test; Visual evoked potential recordings; Electroretinogram (ERG); Visual cliff test
Visual placing reflex<br>(AM1500019)	Response to extend head and forelimb towards a solid object placed in visual field while being suspended by the tail	Visual placing test; Neurological exam battery
Sensory-evoked response: excitation: auditory stimulus<br>(AM1500020)	Activation of sensory neurons (third order neurons or higher) in the cerebral cortex in response to auditory stimuli	Application of auditory stimuli; Measurement of area of activation (ROI) in the insular cortex; Grooming behavior assessments; In vivo local field potential (LFP) recordings
Multisensory integration: sensory neurons of insular cortex: auditory-tactile<br>(AM1500022)	Unification or superimposition of various sensory stimuli, by neurons responsive to multiple types of stimuli, such as tactile, auditory, olfactory or visual	Application of tactile and auditory cues; Quantification of degree of integration
Sensory-evoked response: inhibition: visual stimulus<br>(AM1500023)	Evoked potentials or transient currents associated with inhibition (like chloride ion currents) generated by response to sensory input in sensory neurons, e.g., visual thalamic neurons present in the lateral geniculate nucleus	Chloride photometry
Tactile memory<br>(AM1500024)	Ability to discriminate between different types of surfaces of objects by contact on glabrous skin to establish memory based on tactile perception	Textured novel object recognition test (T-NORT)
Sensorimotor gating: tactile cue<br>(AM1500025)	Process by which inhibitory neural pathways reduce the intensity of an unconditioned response to	Prepulse inhibition

	unfavorable cues that follow training with an conditioning cue, measured by pre-pulse inhibition (PPI) using only an air puff on back hair as the prepulse stimulus for testing hairy skin tactile perception	
Dopaminergic limb retraction reflex (AM1500026)	Slower retraction of hindlimb or forelimb in enclosure with openings for the limbs, in response to administration of a dopamine D1 receptor antagonist, to measure sensitivity to the antagonist	Paw retraction test
Pain or nociception: mechanical (AM1500027)	Perception of pain following exposure to noxious mechanical stimuli, relayed by primary afferent nerve fibers expressed in skin: high threshold C fibers (including 'silent nociceptors')) and slowly adapting Adelta mechanoreceptor fibers. There are several candidate cation channels expressed in these mechanoreceptors that are candidates for giving rise to mechanosensation like ASIC1, 2,3, in addition to TRPV2 and 4, TRPA1 and KCNK channels	Von Frey filament test
Pain or nociception: termal (AM1500028)	Perception of pain following exposure to noxious heat, relayed by primary afferent nerve fibers: unmyelinated C and myelinated Type II A delta nociceptors, as exposure to heat activates their TRPV1 (activated at around 43 degrees celsius) or related TRPV2,3 and 4 channels. These primary afferent nerve fibers project to the dorsal horn of the spinal cord	Paw withdrawal test; Tail flick test; Open field test; Hot plate test; Local inflammatory reaction; Avoidance of noxious heat
Pain or nociception: chemical (AM1500029)	Perception of pain following exposure to noxious chemicals, relayed by primary afferent nerve fibers: unmyelinated C fibers as well as myelinated A delta nociceptors, to the spinal cord. For response to chemical irritants these nociceptors rely on TRP channels including TRPV1, TRMPM8 and TRPA1	
Tail nerve motor conduction (AM1500030)	An action potential transmitted along a single nerve in the tail	Tail action potential recording

Rooting reflex (AM1500035)	Rooting reflex: A reflex seen in normal newborn mammals where the newborn will turn its head toward anything that strokes its face or mouth, searching for the object by moving its head in steadily decreasing arcs until the object is found. Rooting reflex develops before a suckling reflex.	
Vision: phototaxis (AM1500037)	Attraction to light	Fast phototaxis assay; Y-maze test
Visual motor response (AM1500039)	Series of typical motor responses seen in zebrafish in response to ambient illumination, drastic light onset or offset	Optomotor response to dark flash pulses
Startle response (AM1500040)	An action or movement due to the application of a sudden unexpected stimulus.	Light-off startle jump; General observations
Sensory-evoked response: excitation: olfactory stimulus (AM1500041)	Activation of sensory neurons (third order neurons or higher) in the cerebral cortex in response to olfactory stimuli	
Startle response: directional stimulus (AM1500042)	Response of escape swimming to tapping (from one direction) on the walls of the tank, observed in zebrafish larvae, a type of mechanosensory response	
Multisensory integration (AM1500043)	Any neural process required for an organism to sense, integrate and interpret the dimensions of a sensory experience: modality, location, intensity and effect.	
Pain or nociception: thermal: cold (AM1500044)	Perception of pain following exposure to noxious cold, relayed mainly by A delta thin myelinated nerve endings of afferent neurons located deeper in the dermis mediated by TRPA1 and TRPM8 channels, along with Nav1.8, TREK-1 and TRAAK	
**MOTOR**		
**Pheno Term (PhenoID)**	**Pheno Definition**	**Experimental Paradigm**
Ataxia (AM1000000)	Inability to coordinate voluntary muscular movements	General observations; Footprint analysis; Accelerating rotarod test; Dowel walk test; Open field test

Negative Geotaxis (AM1000001)	Reflex in rodents to orient themselves and move away from the force of gravity, tested on a downward or a slanting grid	Negative geotaxis test; Wire hang test; General observations; Neurological exam battery
Clasping reflex (AM1000002)	Clasping of front or hind feet upon being lifted by tail, it is a dystonic posture and is a pathological a reflex that indicates lesions of cerbellum, basal ganglia or neocortex	Tail suspension test; General observations
Climbing (AM1000003)	Behavior of ascending an object, typically a wall	Climbing assay; Open field test; Morris water maze test; Home cage behavior; General observations
Motor learning (AM1000004)	Any process in which an organism acquires a novel neuromuscular action or movement as the result of experience.	Accelerating rotarod test
Gait (AM1000007)	Stride length, paw placement, average speed and posture during walking	Footprint analysis; General observations; Gait; Running stride analysis; Pivoting test; Dowel walk test; Homing test
General locomotor activity (AM1000008)	Free horizontal and vertical movements, including walking, rearing and grooming, usually measured in a controlled chamber using infrared sensors or beam crossing	Open field test; Novel cage test; Elevated plus maze test; General observations; Home cage behavior; Three-chamber social approach test; Reciprocal social interaction test; Running wheel test; Beam crossing; Compartment transitions in three-chambered apparatus; Light-dark exploration test; Novel object recognition test; Conditioned place preference test; Distance traveled in a grid; Observation of repetitive behavior; Shoaling assay; Accelerating rotarod test; Morris water maze test; Disc assay; Object-place recognition test; Wheel running monitoring; Ambulation recordings; Free walking; Wire hang test; Y-maze test; Novel tank diving test
General locomotor activity: Ambulatory activity (AM1000009)	Walking behavior, generally measured by distance traveled or by beam breaks or activation of more than one sensor in an activity monitor cage	Open field test; Home cage behavior; General observations; Elevated plus maze test; Elevated zero maze test; Light-dark exploration test; Three-chamber social approach test; Barnes maze test; Social recognition test; Larval foraging assay; Operant conditioning paradigm; Modified open field test with novel object; Novel object recognition test; Compartment transitions in three-chambered apparatus

Grasping reflex (AM1000010)	Ability of pups to grasp a bar or wire with forelimbs	Neurological exam battery; Horizontal bar test
Grip strength (AM1000011)	Ability to grasp and hold objects, usually measured as time spent hanging from an object or wire	Grip strength test; Wire hang test; Inverted grid test; General observations; Vertical pole test; Neurological exam battery; Ledge test; Accelerating rotarod test; Traction test
Hunched posture (AM1000013)	Posture with body stooped low, the limbs pulled in close to the body and arched back	General observations
Hyperactivity (AM1000014)	General restlessness or excessive movement, or more frequent movement from one place to another, it is a considered a standard response to novel stimulus, including a new environment and is part of non-associative learning	Open field test; Three-chamber social approach test; Novel cage test; Spontaneous movement analysis; Beam crossing; Elevated plus maze test; Home cage behavior; Resident-intruder test; Infrared light beam crossing; Running wheel test; Modified open field test with novel object
Hyperactivity: Home cage/familiar environment (AM1000015)	Increased restlessness or excessive movement in a familiar environment, considered to be reduced non-associative learning	Home cage behavior; Y-maze test
Hypoactivity (AM1000016)	Reduced movements and activity, similar to a sedative state caused by drug or change in emotional status	Elevated plus maze test; General observations; Open field test
Jerky Movement (AM1000018)	Continuous, abrupt, faltering motions of the whole body or a portion of the body	General observations; Tail suspension test; Observation of repetitive behavior
Limb Posture (AM1000020)	Position of the limbs or carriage of the body	General observations; Tail suspension test
Motor coordination and balance (AM1000021)	Ability to execute integrated movements of muscle	Accelerating rotarod test; Balance beam test; Wire hang test; Dowel walk test; General observations; Marble-burying test; Footslip test; Fixed bar test; Vertical pole test; Parallel bar test; Traction test; Motion-induced landing; Footprint analysis; Erasmus ladder test; Cylinder test; Horizontal bar test; Horizontal ladder test; Open field test
Motor strength and endurance (AM1000022)	A summarized measurement for body muscle strength and endurance, including observations for hypotonia	Dowel walk test; Neurological exam battery; Progressive treadmill test; Vertical pole test; Accelerating rotarod test; Wire hang test
Paw preference (AM1000024)	Behavioral inclination to use either the right or left paw in a task	Dynamic weight bearing; Catwalk; Paw preference test

Postural reflex (AM1000026)	Responses that control the normal position of the trunk and extremities or posture	General observations; Righting reflex test; Tail suspension test; Upright balance test
Righting response (AM1000027)	Ability to recover from supine position to prone position	Righting reflex test; General observations; Negative geotaxis test; Neurological exam battery; Cliff avoidance test
Self grooming: home cage/familiar environment (AM1000028)	A complex innate behaviour with an evolutionary conserved sequencing pattern observed in all rodents, it is required for several physiologically important processes:hygiene maintenance, thermoregulation, social communication and de-arousal. It is one of the most frequently observed behaviours in awake rodents with characteristic head-to-body progression and also comprises predictable synctatic chain patterns as well as flexible (non predictable) sequential patterning	Home cage behavior; Observation of repetitive behavior; Grooming behavior assessments; General observations; Object-place recognition test
Spinal reflex (AM1000029)	Reflexive action mediated by cells of the spinal cord, or the involuntary movement reaction caused by the application of a stimulus to an organism	Knee joint reflex test; General observations
Spontaneous and reflexive eye movements (AM1000030)	Spontaneous eye movements and reflexive movements like blinking, in response to stimulation in or around the eye area e.g., corneal reflex; maybe associated with some cerebellar, spinal cord or medullar dysfunction	Eye movement recording; Corneal reflex test
Spontaneous Movement (AM1000031)	Spontaneous change in position or posture, without external stimulus	Spontaneous movement analysis; Open field test; Home cage behavior; General observations
Swimming ability (AM1000032)	Ability to swim well, often determined by swimming speed	Morris water maze test; Swim test; General observations; Forced swim test; Water Y-maze; Shoaling assay; Stereology
Tremor (AM1000033)	Involuntary or uncontrollable muscle movement	General observations; Tremor activity measurements; Electromyogram (EMG)
Walking backwards (AM1000035)	Locomotor activity in the posterior direction	General observations

Self grooming: artificial stress evoked (AM1000036)	Grooming in response to induced stress by a splash of sucrose solution (splash test), intended for hygiene or de-arousal	Grooming behavior assessments; Splash test to evoke grooming behavior
General locomotor activity: Larval locomotor behavior (AM1000037)	Crawling behavior of fly larva	General observations; Sleep analysis
Swimming ability: spontaneous coiling (AM1000038)	Spontaneous coiling occurs at approximately 17 hpf and is the first stage of swimming locomotor development characterized by spontaneous alternating lateral trunk flexion with no body displacement.	General observations
Swimming ability: evoked coiling (AM1000039)	Evoked coiling appears approximately at 21 hpf and is the second locomotor stage in zebrafish charecerized by sensory driven movement in which manual stimulation of the embryo elicits alternating lateral trunk flexion with no displacement.	General observations
Swimming ability: burst swimming (AM1000040)	The third stage of locomotor development, known as burst swimming, begins at approximately 27 hpf. Larvae lie on their side at the bottom of the petridish and show little volitional movement. When larvae are touched, they will rapidly swim to the opposite side of the petri dish and lie once again on their side.	General observations
Swimming ability: beat swimming (AM1000041)	Beat swimming is a characteristic of the final stage of locomotory development (spontaneous swimming activity) in zebrafish and is characterized by coordinated volitional movement after inflation of the swim bladder. At this stage, both speed and direction of movement can be controlled.	General observations
Swimming ability: glide swimming (AM1000042)	Glide swimming is a characteristic of the final stage of locomotory development (spontaneous swimming activity) in zebrafish and is characterized by coordinated volitional movement after inflation of the	General observations

	swim bladder. At this stage, both speed and direction of movement can be controlled.	
General locomotor activity: flight (AM1000043)		Flight performance test
Ataxia: turn angle (AM1000044)	The angle at which larvae make turns during movement	Video Recordings
Motor coordination and balance: fine motor skills (AM1000045)	Fine motor skill or dexterity is the coordination of small muscles in movements, usually involving the synchronisation of the musculoskeletal and sensory system.	
